# Structural basis for impaired 5′ processing of a mutant tRNA associated with defects in neuronal homeostasis

**DOI:** 10.1073/pnas.2119529119

**Published:** 2022-03-01

**Authors:** Lien B. Lai, Stella M. Lai, Eric S. Szymanski, Mridu Kapur, Edric K. Choi, Hashim M. Al-Hashimi, Susan L. Ackerman, Venkat Gopalan

**Affiliations:** ^a^Department of Chemistry & Biochemistry, The Ohio State University, Columbus, OH 43210;; ^b^Center for RNA Biology, The Ohio State University, Columbus, OH 43210;; ^c^Department of Biochemistry, School of Medicine, Duke University, Durham, NC 27710;; ^d^Department of Cellular and Molecular Medicine, Section of Neurobiology, University of California San Diego, La Jolla, CA 92093;; ^e^Howard Hughes Medical Institute, University of California San Diego, La Jolla, CA 92093

**Keywords:** tRNA processing, neurodegeneration, conformational toggling, tRNA-Arg-TCT-4-1

## Abstract

*n-Tr20* is a neuron-specific, cytoplasmic transfer RNA^Arg^_UCU_ (tRNA^Arg^_UCU_) isodecoder that affects seizure susceptibility, neuronal excitability, and translation signaling in mice. In addition, the C50U substitution in *n-Tr20* (*n-Tr20^C50U^*), which is found in the widely used C57BL/6J (B6J) inbred mouse line, contributes to neurodegeneration by epistatic interactions with mutations in *Gtpbp1* or *Gtpbp2*, two factors that rescue ribosomal stalling. The brains of B6J mice have high levels of immature and low levels of mature *n-Tr20^C50U^*, implicating defective tRNA biogenesis as the basis for neuronal dysfunction, a hypothesis tested in this study. We demonstrate that partially purified mouse brain ribonuclease P, the endonuclease responsible for 5′ maturation of tRNAs, exhibits at 1 mM magnesium a 20-fold lower apparent cleavage rate for processing the *n-Tr20^C50U^* precursor than the wild-type precursor. Thermal denaturation studies unexpectedly revealed that substituting the native C_50_-G_64_ base pair with the U_50_-G_64_ wobble pair in *n-Tr20^C50U^* increased the T_m_ by as much as 8 °C, with the magnitude of the change dependent on [Mg^2+^]. Moreover, results from thermal denaturation, native gel electrophoresis, ^1^H nuclear magnetic resonance spectroscopy, and kinetic studies collectively support the presence of stable alternative folds for *n-Tr20^C50U^* that may also be sampled transiently and in low abundance by the wild-type tRNA. These findings suggest that mutation-driven restructuring could foster nonnative folds and that conformational toggling of tRNAs poses an intrinsic risk for dysfunction when point mutations selectively stabilize nonnative states. This model may be applicable for understanding the molecular basis of other diseases that are associated with tRNA mutations.

## Significance

Understanding and treating neurological disorders are global priorities. Some of these diseases are engendered by mutations that cause defects in the cellular synthesis of transfer RNAs (tRNAs), which function as adapter molecules that translate messenger RNAs into proteins. During tRNA biogenesis, ribonuclease P catalyzes removal of the transcribed sequence upstream of the mature tRNA. Here, we focus on a cytoplasmic tRNA^Arg^_UCU_ that is expressed specifically in neurons and, when harboring a particular point mutation, contributes to neurodegeneration in mice. Our results suggest that this mutation favors stable alternative structures that are not cleaved by mouse ribonuclease P and motivate a paradigm that may help to understand the molecular basis for disease-associated mutations in other tRNAs.

**Fig. 1.**
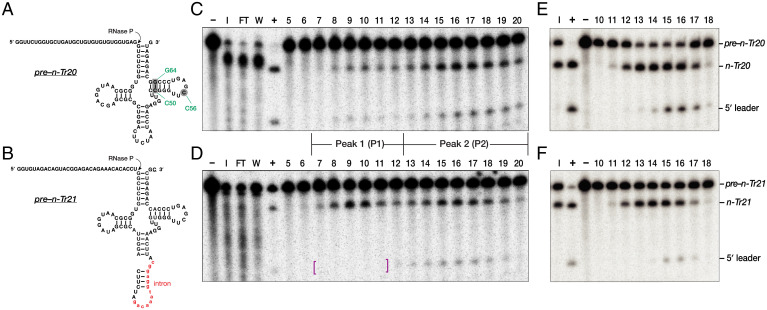
Purification of mouse brain RNase P. (*A*, *C*, and *E*) *Pre–n-Tr20* and (*B*, *D*, and *F*) *pre–n-Tr21* were used to assay individual purification fractions for RNase P activity. While both pre-tRNAs contain a 32-nt 5′ leader, *pre–n-Tr21* also has a 13-nt intron (red lowercase) and a 1-nt 3′ trailer (underlined). The arrows indicate the expected RNase P cleavage site. In *A*, the three positions that are mutated in different *n-Tr20* variants used in this study are shown in green. (*C* and *D*) RNase P assays of the elution fractions from the DEAE column. In *D*, magenta-colored brackets are used to highlight degradation of the cleaved 5′ leader of *pre–n-Tr21* in fractions 7 to 11. The two reproducible peaks of activity are indicated: P1 (fractions 7 to 12) and P2 (fractions 13 to 20). (*E* and *F*) Further purification of P1 using a 10 to 35% (vol/vol) glycerol gradient. Fractions 14 to 16 were pooled to yield P1F (the final P1 preparation). Lanes are as follows: I, input; FT, flow through; W, wash; –, negative control containing substrate but no enzyme; +, positive control with substrate and in vitro–reconstituted *E. coli* RNase P (49).

Transfer RNAs (tRNAs) are the most highly expressed, small noncoding RNAs in all domains of life (1, 2). While many diseases arise from mutations in processing/modifying enzymes that act on multiple tRNAs (3–5), mutations in single tRNA genes in higher eukaryotes have largely been overlooked as drivers of disease due to the multiplicity of genes encoding tRNAs with the same anticodon (isodecoders) and the expectation that these RNA polymerase III–transcribed genes are ubiquitously expressed. However, there is growing evidence that the expression of individual tRNAs can vary between tissues and with cellular state, stress, and disease (6–11). In this regard, *n-Tr20* (Fig. 1*A*) and its human ortholog (*tRNA-Arg-TCT-4-1*), members of the cytoplasmic *tRNA-Arg-TCT* family (five genes in mice and six in humans), we found to be tissue-specifically expressed. *n-Tr20* is specifically expressed in neurons where it is the most prominently expressed member of this tRNA family (7, 12, 13). Moreover, a mutant form of *n-Tr20* contributes to neurodegeneration (7, 12, 14).

The C50U mutation in *n-Tr20* (*n-Tr20^C50U^*) found in the C57BL/6J (B6J) inbred mouse strain leads to an accumulation of immature *n-Tr20^C50U^* with a 5′ leader and a 3′ trailer, which are normally removed during tRNA maturation in preparation for aminoacylation. Interestingly, this mutation has also been found in a human Arg-TCT tRNA (15). Accumulation of this immature tRNA was accompanied by a corresponding decrease in the level of mature aminoacylated tRNA and, ultimately, ribosome stalling at AGA codons (7). Homozygosity for this mutation resulted in neurodegeneration when associated with the loss of guanosine-5'-triphosphate (GTP) binding protein 1 (GTPBP1) or GTPBP2, which relieves ribosome stalling (7, 14). Both B6J.*Gtpbp1*^−/−^ and B6J-*Gtpbp2*^−/−^ mice exhibit pronounced truncal ataxia starting at 4 wks of age, concomitant with apoptosis of neurons in the inner granule layer of the cerebellum, as well as degeneration of hippocampal, cortical, and retinal neurons. This neurological phenotype results from a breach in the GTPBP1/2-mediated translational surveillance mechanism; ribosome profiling revealed that *n-Tr20^C50U^*–induced stalling at AGA codons increased in the absence of these proteins (7, 14). Furthermore, loss of *n-Tr20* expression (caused by either the C50U mutation or knocking out *n-Tr20*) is sufficient to confer resistance to electrically and pharmacologically induced seizures (12). *n-Tr20*^C50U^ homozygosity also modified the effect of an epilepsy-associated mutation in the gamma-aminobutyric acid_A_ receptor γ2-subunit as evidenced by a decreased frequency of spike-wave discharges (12). Decreased levels of *n-Tr20* represses translation and activates the integrated stress response via general control nonderepressible 2, a protein kinase that is activated by ribosomal stalling (12).

The above studies, which unmasked the disease potential of tRNA genes, motivated further biochemical and structural characterization of *n-Tr20*. Here, we sought to dissect the molecular basis for defective processing of *n-Tr20^C50U^* that has a U_50_-G_64_ base pair (bp) instead of a C_50_-G_64_ bp in the T stem (7). Since most cytoplasmic precursor tRNAs (pre-tRNAs) are processed in the 5′-before-3′ order in eukaryotes (16, 17) and the brains of B6J mice accumulated a 105-nucleotide (nt) immature *n-Tr20^C50U^* with a 5′ leader and a 3′ trailer instead of the 77-nt mature *n-Tr20^C50U^* (7), we hypothesized that the C50U mutation impairs cleavage by ribonuclease (RNase) P, the endonuclease that removes the 5′ leader of pre-tRNAs (18–22). Although the composition [one catalytic RNA and ≤10 protein cofactors,] and cryogenic electron microscopy structures [that reveal subunit arrangement and substrate–recognition determinants (23, 24)] of eukaryotic nuclear RNase P are known, it is difficult to explain why this mutation that is distal to the RNase P cleavage site would adversely affect 5′ processing. Moreover, U_50_ is not uncommon in tRNAs (25) and is even present in two other Arg-TCT isodecoders, *n-Tr21* (*tRNA-Arg-TCT-3-1*) (Fig. 1*B*) and *n-Tr25* (*tRNA-Arg-TCT-2-1*). Our thermal denaturation, native gel electrophoresis, nuclear magnetic resonance (NMR) spectroscopy, and kinetic studies of wild-type (WT) and mutant *n-Tr20* led to the surprising finding that *n-Tr20^C50U^* adopts stable nonnative structures. These results form the basis for a conformational toggling model that posits how intrinsic vulnerabilities associated with tRNA folding are amplified by point mutations.

## Results

### Purification of RNase P from Mouse Cortex.

To determine whether the C50U mutation affects 5′ processing of *pre–n-Tr20^C50U^*, we partially purified RNase P from the brain cortices of B6J mice. The cortical lysate was loaded on a diethylaminoethyl (DEAE) (anion-exchange) column, and the eluted fractions were assayed for RNase P activity with two in vitro–transcribed WT pre-tRNA^Arg^_UCU_ isodecoders: *pre–n-Tr20* and *pre–n-Tr21*, both with a 5′ leader but only the latter had a single-nt 3′ trailer (Fig. 1 *A* and *B*). We consistently observed two reproducible and separable peaks (P) of RNase P activity. Because these two biochemically distinct forms could potentially process *pre–n-Tr20* and –*n-Tr20^C50U^* differently, active fractions from each peak were pooled separately as P1 and P2 (Fig. 1 *C* and *D*).

Both P1 and P2 activities were unstable and steadily lost activity within a week of purification, likely due to degradation of the RNase P RNA by coeluting nucleases (Fig. 1*D*). Therefore, P1 was further purified using a 10 to 35% (vol/vol) glycerol gradient (Fig. 1 *E* and *F*) and then concentrated sevenfold using a centrifugal filter. While P2 lost all activity when subjected to further purification, its activity could be prolonged when concentrated 10-fold. The final preparations, P1F and P2F, had a longer shelf-life and lasted about a month. Importantly, both activities catalyzed site-specific cleavage of *n-Tr20* (*SI Appendix*, Fig. S1).

### The 3′ Trailer Does Not Interfere with 5′ Processing of *Pre*–*n-Tr20* by Mouse Brain RNase P.

The presence of a 3′ trailer in pre-tRNAs has no effect on cleavage by eukaryotic nuclear RNase P (16) (an exception is in ref. 26). However, it was unclear whether this attribute holds for *n-Tr20^C50U^* because B6J brains accumulated an immature *n-Tr20^C50U^* with both a 5′ leader and a 3′ trailer (7). Therefore, we assayed P1F and P2F for their ability to cleave in vitro–transcribed *pre–n-Tr20 + 3′* containing a 5′ leader and a 3′ trailer (*SI Appendix*, Fig. S2). P1F and P2F cleaved the 5′ leader of both *pre–n-Tr20 + 3′* and *pre–n-Tr20^C50U^+3′*, albeit the latter was much poorer, revealing that it is the C50U mutation, not the trailer, that hampers RNase P cleavage. In addition to this 5′ maturation activity, we observed that the 3′ trailer was also cleaved in our assays due to a coeluting 3′ processing activity (likely RNase Z) in both P1F and P2F (*SI Appendix*, Fig. S2). Notably, the pattern of product formation indicated that 3′ processing took place only with the RNase P–processed intermediate, consistent with previous reports that most eukaryotic pre-tRNAs are cleaved by RNase P before RNase Z (17).

### Mg^2+^-Dependent Cleavage Rates of Mouse Brain RNase P Vary with Substrate Identity.

**Fig. 2.**
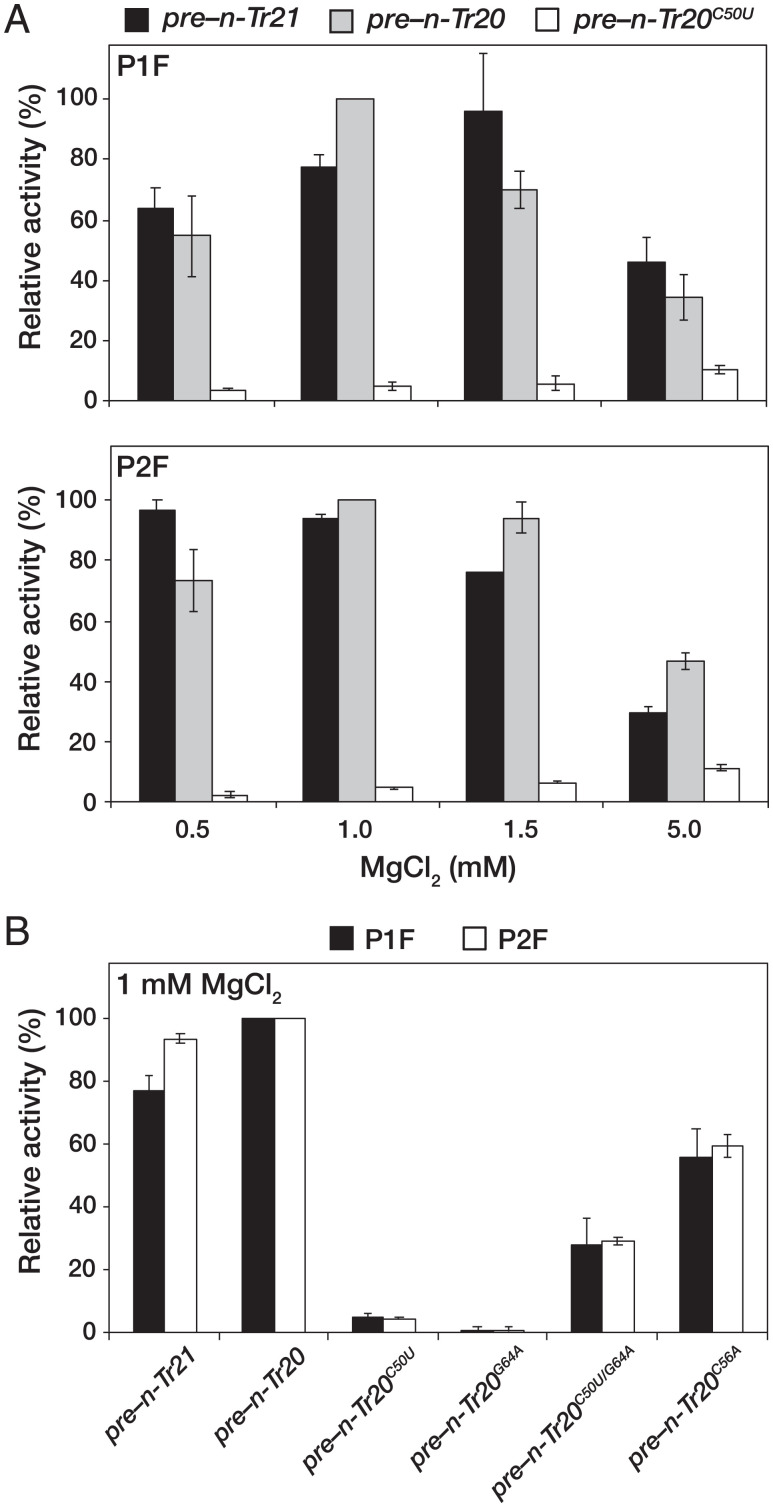
Comparison of the apparent cleavage rates of different pre-tRNAs by mouse brain RNase P. (*A*) Relative Mg^2+^-dependent cleavage activities for processing *pre–n-Tr21*, *pre–n-Tr20*, and *pre–n-Tr20^C50U^* by the two peaks of RNase P activity, P1F (*Upper*) and P2F (*Lower*). (*B*) Relative cleavage activities for processing *pre–n-Tr21*, *pre–n-Tr20*, and different mutant derivatives of *pre–n-Tr20* in 1 mM MgCl_2_. All values are normalized to the activity observed with *pre–n-Tr20*. Mean and standard deviation values were calculated from three independent initial velocity measurements.

Site-specific coordination of Mg^2+^ in binding pockets (particularly the elbow) of tRNAs is critical for their overall three-dimensional architecture which, in turn, is important for function (e.g., aminoacylation) (27–29). We hypothesized that the C50U mutation may weaken the T stem and consequently, tertiary contacts in *n-Tr20^C50U^*. Therefore, we tested P1F and P2F for their ability to cleave *pre–n-Tr21*, *–n-Tr20*, and *–n-Tr20^C50U^* (all with only a 5′ leader) in 0.5, 1, 1.5, or 5 mM MgCl_2_. P1F and P2F displayed slightly different MgCl_2_ optima during cleavage of *pre–n-Tr21* and –*n-Tr20*, with maximal activity in near-physiological [MgCl_2_] (Fig. 2*A*). P2F also appeared to be less sensitive than P1F to [MgCl_2_] changes between 0.5 and 1.5 mM. Interestingly, *pre–n-Tr20^C50U^* was cleaved poorly by both P1F and P2F regardless of the [MgCl_2_] tested, with minimal rescue even in 5 mM MgCl_2_ (Fig. 2*A*). While their differential elution from DEAE is suggestive of differences in the compositional makeup of P1F and P2F, both cleaved *pre–n-Tr20^C50U^* with an apparent cleavage rate that is 20-fold slower than the WT in 1 mM MgCl_2_, the optimal concentration for processing *pre–n-Tr20* (Fig. 2). Despite this weak cleavage, processing fidelity was maintained with the mutant pre-tRNA (*SI Appendix*, Fig. S1).

To investigate whether tissue-specific factors in the mouse brain RNase P preparations somehow dampen the enzyme’s ability to process specific mutant pre-tRNAs, we partially purified RNase P from the livers of 4-wk-old B6J mice and conducted a cleavage assay with *pre–n-Tr20* and *–n-Tr20^C50U^* at 1 and 5 mM MgCl_2_. Again, we observed a profound decrease in *pre–n-Tr20^C50U^* processing (*SI Appendix*, Fig. S3), suggesting that this cleavage defect arises from structural perturbations in *pre–n-Tr20^C50U^* rather than tissue-specific variations in RNase P.

### The N_50_-N_64_–bp Identity in *n-Tr20* Influences the Rate of RNase P Cleavage.

While the C50U mutation does not eliminate base pairing (C_50_-G_64_ is replaced by U_50_-G_64_), poor cleavage of *pre–n-Tr20^C50U^* by both P1F and P2F prompted us to examine the importance of the identity of N_50_ and N_50_-N_64_ bp itself. Therefore, we constructed two mutant derivatives and examined their cleavage by P1F and P2F in 1 mM MgCl_2_. *Pre–n-Tr20^G64A^* (with a C_50_-A_64_ mismatch) was processed at only 1% of the apparent cleavage rate observed for *n-Tr20* (Fig. 2*B*), suggesting that the N_50_-N_64_ bp is important for promoting a pre-tRNA structure recognized by RNase P. Surprisingly, despite the isosteric replacement of C_50_-G_64_ with U_50_-A_64_, the apparent cleavage rate for *pre–n-Tr20^C50U/G64A^* was only ∼30% of that determined for *pre–n-Tr20* (Fig. 2*B*). These data indicate a preference for a C_50_-G_64_ bp in *n-Tr20* to promote an optimal structure for recognition and cleavage by RNase P. Although the identity of N_50_ may not be an absolute determinant for mouse RNase P activity (as supported by the robust cleavage of *pre–n-Tr21*, which has a U_50_-A_64_ bp) (Fig. 1*B*), it is conceivable that substitution of C_50_ with U_50_ in *n-Tr20* alters its conformational ensemble (see below).

### *n-Tr20^C50U^* Is More Stable than WT *n-Tr20*.

**Fig. 3.**
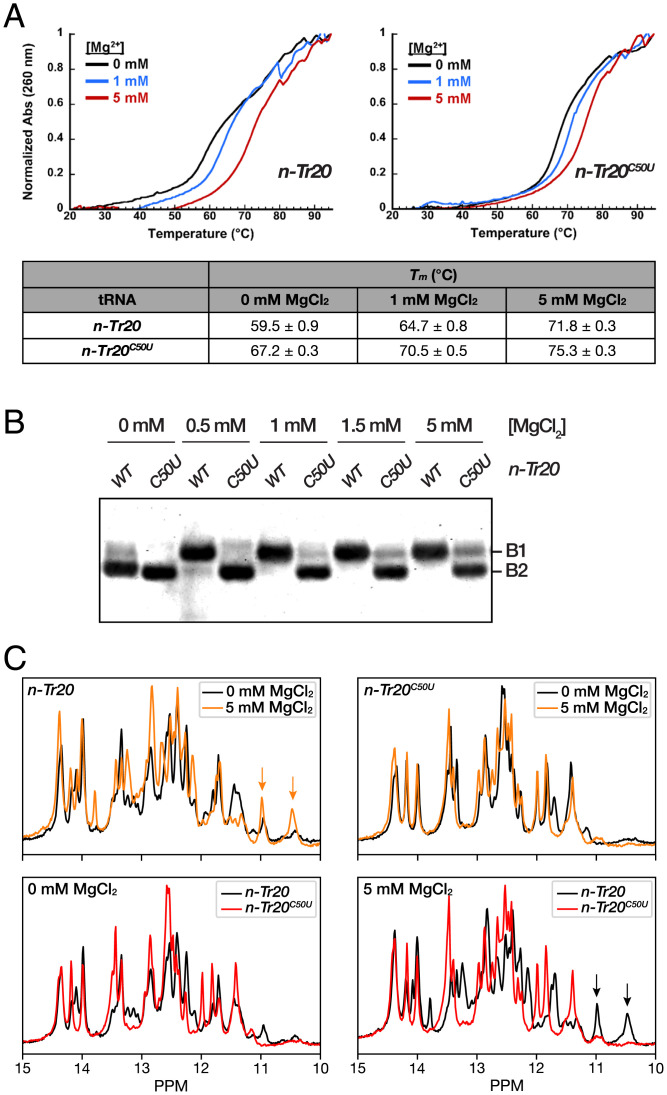
Structural characterization of mature *n-Tr20* and *n-Tr20^C50U^*. (*A*) Thermal denaturation experiments reveal increased structural stability conferred by the C50U mutation. (*Upper*) Normalized absorbance at 260 nm is plotted as a function of increasing temperature in 0, 1, or 5 mM MgCl_2_. Mean and SD values were calculated from three independent thermal melts. (*B*) Native PAGE analyses reveal Mg^2+^-dependent tRNA conformational changes. The two tRNAs migrated as two bands, labeled B1 and B2, with B1 likely constituting the native fold. (*C*) The imino proton region of ^1^H NMR spectra of *n-Tr20* and *n-Tr20^C50U^* exhibits two distinct conformers, with *n-Tr20* showing more Mg^2+^-induced alterations than *n-Tr20^C50U^*. (*Upper*) Overlaid spectra for each tRNA in 0 vs. 5 mM MgCl_2_. (*Lower*) Comparison of the two tRNAs in 0 vs. 5 mM MgCl_2_. Orange and black arrows indicate the two upfield peaks in *n-Tr20* that form in the presence of MgCl_2_ and that likely indicate tertiary contacts (*SI Appendix*, Figs. S6 and S7).

We used thermal denaturation experiments to investigate the stability of in vitro–transcribed mature *n-Tr20* and its mutant derivatives and to gain insights into whether the observed differences in the apparent RNase P cleavage rates of these substrates reflect their respective distribution between “cleavable” and “noncleavable” conformers (28, 30). Monitoring hyperchromicity as a function of increasing temperature traces the transition from a folded to an unfolded state, and the resulting T_m_ values can provide insights into the relative stabilities of different RNAs (28). For tRNAs, the nuanced low-temperature transition (∼40 °C) is typically attributed to unraveling of the elbow (tertiary contacts) (30), while a sharper higher-temperature transition corresponds to cooperative melting of the secondary structure (28, 31). Thermal denaturation experiments were conducted in 0, 1, or 5 mM MgCl_2_ to assess whether potential nonnative structural realignments in tRNA mutants might be reversed by increasing [MgCl_2_]. Normalized absorbance at 260 nm was plotted vs. temperature (20 to 95 °C) (Fig. 3*A*), and the corresponding first-derivative plots (dA/dT vs. temperature) (Fig. 3*A*) were generated. The T_m_ values for each tRNA were obtained from the maxima of the first-derivative plots.

Based on previous studies that examined disease-associated mutant tRNAs with single-nt substitutions (32, 33), the C50U mutation was expected to decrease the T_m_ of *n-Tr20*. However, we were surprised to find that *n-Tr20^C50U^* exhibited a T_m_ that was ∼8 °C higher than the WT in the absence of MgCl_2_ (Fig. 3*A*). Increasing [MgCl_2_] from 0 to 5 mM raised the T_m_ by 12 °C for *n-Tr20* and 8 °C for *n-Tr20^C50U^* (Fig. 3*A*). While the T_m_ observed with *n-Tr20^C50U^* remained higher than *n-Tr20* in all three MgCl_2_ concentrations, the rift narrowed with increasing [MgCl_2_] (Fig. 3*A*). Remarkably, the compensatory base substitution mutant *n-Tr20^C50U/G64A^* also displayed increased stability, with similar T_m_ values as *n-Tr20^C50U^* in 0, 1, and 5 mM MgCl_2_ (*SI Appendix*, Fig. S4). The C_50_-A_64_ mismatch mutant *n-Tr20^G64A^* had T_m_ values between those of *n-Tr20* and *n-Tr20^C50U^*/*n-Tr20^C50U/G64A^*, suggesting that *n-Tr20^G64A^* adopts a secondary structure that is distinct from the other tRNAs.

### Conformational Sampling by *n-Tr20* and Its Mutants.

Our unexpected finding of the increased structural stability of native *n-Tr20^C50U^* prompted us to examine the conformational attributes of all in vitro–transcribed variants of mature *n-Tr20* using native polyacrylamide gel electrophoresis (PAGE), which allows for the visualization and quantitation of multiple conformations with distinct electrophoretic mobilities (34). In 0 mM MgCl_2_, we detected two species for *n-Tr20*: a slower-migrating band (B1) that is much fainter than a faster-migrating band (B2) (Fig. 3*B*). Consistent with the expectation that *n-Tr20* attains a native-like fold at higher [MgCl_2_], the proportion of *n-Tr20* found in B1 increased with increasing [MgCl_2_], while B2 disappeared completely by 1 mM MgCl_2_. Thus, B1 likely reflects the native conformation. *n-Tr20^C50U^* migrated as a single band (B2) in 0 mM MgCl_2_. The slight increase in the amount of B1 from 0.5 to 5 mM MgCl_2_ suggests that only a small fraction of *n-Tr20*^C50U^ attains a native-like fold even in 5 mM MgCl_2_ (Fig. 3*B*), in sharp contrast to the trend observed with *n-Tr20*.

The other *n-Tr20* mutants also showed interesting conformational differences. Like *n-Tr20^C50U^*, *n-Tr20^C50U/G64A^* and *n-Tr20^G64A^* migrated only as B2 in 0 mM MgCl_2_ (*SI Appendix*, Fig. S5). More than half of *n-Tr20^C50U/G64A^* shifted to B1 in 1 mM MgCl_2_, with a further shift in 5 mM MgCl_2_. While MgCl_2_ also induced a slower migration for *n-Tr20^G64A^*, the shift seemed incremental and was more discernible in 5 mM MgCl_2_, where *n-Tr20^G64A^* existed in two bands that appeared to migrate between B1 and B2. As observed in the thermal denaturation curves, *n-Tr20^G64A^* behaved differently from *n-Tr20*, *n-Tr20^C50U^*, and *n-Tr20^C50U/G64A^*. Interestingly, the native PAGE migration profiles for all *n-Tr20* mutants reflected roughly their cleavability by RNase P (Fig. 2*B* and *SI Appendix*, Fig. S5).

To fingerprint the putative alternative secondary structure of *n-Tr20^C50U^* predicted by thermal denaturation and native PAGE analyses, we performed one-dimensional (1D) ^1^H solution-state NMR experiments on all the *n-Tr20* variants described above. The imino protons of G-H1 and U-H3 are excellent reporters of the secondary structure as they depend on the nature of base pairing (35). Experiments were performed at varying MgCl_2_ concentrations (0, 0.5, 1, 2, 5, or 10 mM) to assess RNA folding (Fig. 3*C* and *SI Appendix*, Fig. S6). The 1D ^1^H spectra of *n-Tr20* showed many overall changes with increasing [MgCl_2_], with some already apparent even between 0 and 0.5 mM MgCl_2_, most notably the stabilization of two resonances at ∼11 and ∼10.5 ppm (Fig. 3*C* and *SI Appendix*, Fig. S6). Although unassigned, these distinct upfield peaks are consistent with Mg^2+^-stabilized tertiary contacts between the T and D loops in the tRNA elbow (36). In contrast, the spectra of *n-Tr20^C50U^* changed very little with increasing [MgCl_2_] as high as 10 mM (*SI Appendix*, Fig. S6), which is consistent with results from native PAGE analyses (Fig. 3*B*). The absence of the upfield peaks at ∼11 and ∼10.5 ppm suggested the lack of T loop–D loop interactions. Further differences throughout the spectra of *n-Tr20^C50U^* relative to *n-Tr20* (Fig. 3*C*) suggest broad secondary structural changes that involve multiple G-C bps, at least one A-U bp, and the possible formation of one or two new G-U wobble pairs.

Next, we obtained 1D ^1^H NMR spectra for *n-Tr20^G64A^* and *n-Tr20^C50U/G64A^* in 1 mM Mg^2+^. Like *n-Tr20^C50U^*, both *n-Tr20^G64A^* and *n-Tr20^C50U/G64A^* lack the upfield peaks at ∼11 and ∼10.5 ppm. Consistent with the observations made with thermal melt and native PAGE analyses, the imino spectrum of *n-Tr20^G64A^* shows a unique pattern that is unlike that of *n-Tr20* or *n-Tr20^C50U^* (*SI Appendix*, Fig. S7). In contrast, the spectrum of *n-Tr20^C50U/G64A^* appeared to result from an ensemble resembling partly that of *n-Tr20* and *n-Tr20^C50U^* and suggested that the U_50_-A_64_ bp does not stabilize the structure to the same extent as the native G_50_-C_64_ bp (*SI Appendix*, Fig. S7).

Our hypothesis that C50U destabilizes tertiary contacts prompted us to construct the *n-Tr20*^C56A^ mutant, expecting that it would provide a spectral signature indicative of a destabilized elbow. C56, the third position in the penta-nt T loop, forms a canonical bp with G19 in the D loop (29). However, we found that the imino spectrum of *n-Tr20^C56A^* largely resembled that of *n-Tr20* including the two upfield peaks ∼11 and ∼10.5 ppm (*SI Appendix*, Fig. S7), suggestive of similar structures, an inference further supported by their concordant T_m_ values and similar behavior in native PAGE (Fig. 3*A* and *SI Appendix*, Figs. S4 and S5). Thus, the C56A mutation alone may be inadequate to completely disrupt the tertiary brace that is also buttressed by the intercalation of D-loop G18 between T-loop G57 and A58 to create a contiguous stack of 5 nt (37). Nevertheless, since *pre–n-Tr20^C56A^* is cleaved by RNase P approximately twofold slower than *n-Tr20* (Fig. 3*B*), tertiary interactions must be weakened in *n-Tr20^C56A^*, and conformational switching is likely, as supported by native PAGE where its B2 band was detectable even in 5 mM MgCl_2_ (*SI Appendix*, Fig. S5).

### A Model Depicting Structural Toggling of *n-Tr20* and Its Mutant Derivatives.

**Fig. 4.**
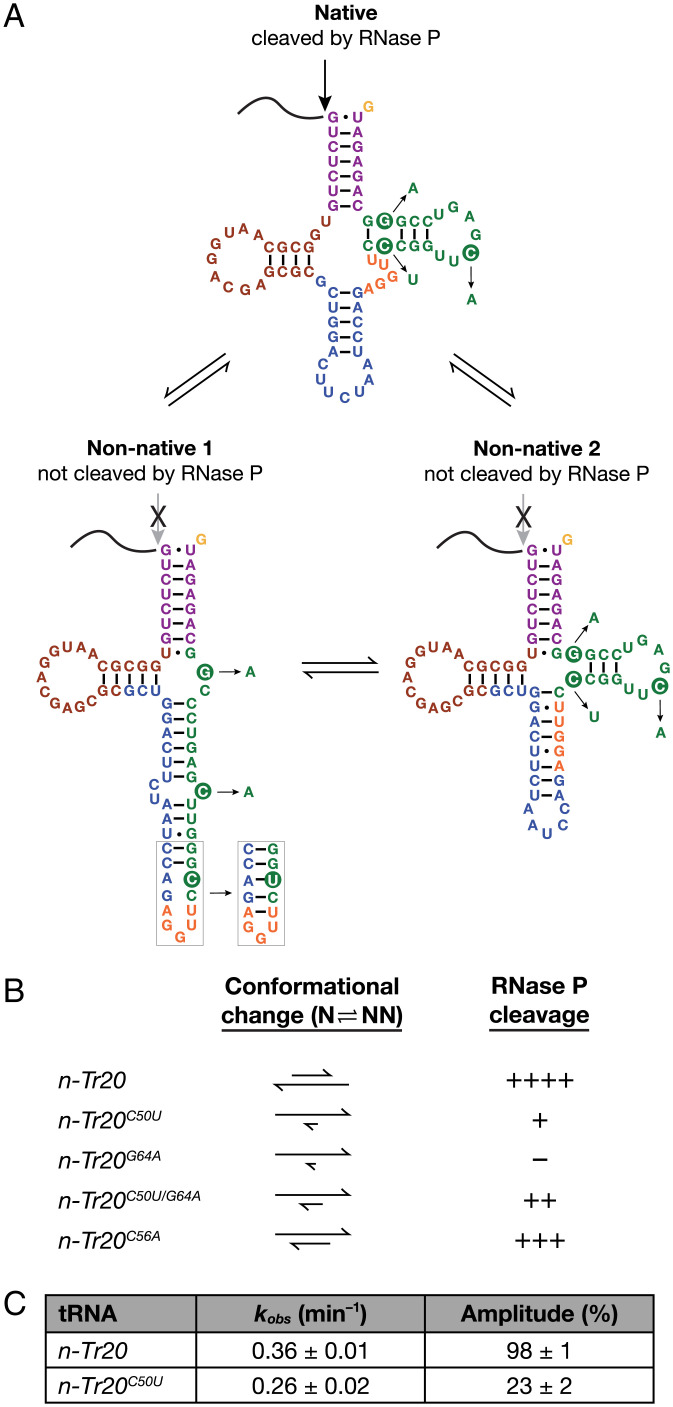
Postulated nonnative conformers of *n-Tr20* and its mutant derivatives. (*A*) *n-Tr20* may toggle between the native (favored in the presence of Mg^2+^) and several nonnative conformations, including the two shown here. Both nonnative configurations likely lack the native tRNA elbow, a key recognition determinant necessary for RNase P cleavage (22–24). (*B*) The propensity of mouse RNase P to cleave each *n-Tr20* variant (data are from Fig. 2*B*) likely reflects the proportion of tRNA that adopts the native conformation. Cleavage efficiency: ++++, fully competent; +/++/+++, partially competent to varying extents; –, defective. N, native structure; NN, nonnative structure. (*C*) Summary of data from single-turnover kinetic studies conducted with in vitro–reconstituted *E. coli* RNase P in 5 mM Mg^2+^ and at pH 5.5. Mean and standard deviation values were determined from three independent measurements.

While we expected the C50U mutation to be destabilizing, it actually increased the T_m_ of *n-Tr20^C50U^* by ∼4 to 8 °C relative to that of *n-Tr20* (Fig. 3*A*). A more compact *n-Tr20^C50U^* structure would also be consistent with the faster mobility observed in native PAGE (Fig. 3*B*). In addition to an RNAFold prediction (http://gtrnadb.ucsc.edu/genomes/eukaryota/Mmusc39/genes/tRNA-Arg-TCT-4-1.html), we also considered an alternative fold for *n-Tr20^C50U^* (nonnative 2) (Fig. 4*A*). Both nonnative folds have more bp and disrupted tertiary contacts compared with the canonical structure predicted for *n-Tr20*. While additional data are needed to confirm the assignment of individual resonances in the NMR spectra and to obtain a high-resolution tertiary structure, the spectral “fingerprints” for *n-Tr20* mutants (Fig. 3*C* and *SI Appendix*, Figs. S6 and S7) support the presence of nonnative secondary structures that lack the tertiary contacts required to form the tRNA elbow in *n-Tr20^C50U^* and other variants. There is a clear correlation between this conformational switching and the apparent rate of cleavage for each tRNA by mouse RNase P (Fig. 4*B*), despite some minor discrepancies likely due to the differences in the conditions used for RNase P assays, thermal melts, native PAGE, and NMR.

As an independent test of the conformational toggling idea, we performed single-turnover kinetic studies where [enzyme] ≫ [substrate]. If *n-Tr20* mostly adopts a conformation that is cleavable by RNase P while *n-Tr20^C50U^* samples two or more conformations and only one of which coincides with the predominantly native conformation assumed by *n-Tr20*, then the *k*_obs_ values for processing *n-Tr20* and *n-Tr20^C50U^* should be similar under single-turnover conditions; moreover, the maximal product formation should be lower for the mutant tRNA and proportional to the fraction of *n-Tr20^C50U^* that adopts the RNase P–cleavable conformation. Because we sought to test a substrate structural attribute and needed a firm knowledge of the enzyme concentration for the single-turnover kinetic studies, we used recombinant bacterial RNase P. In 5 mM Mg(OAc)_2_ and at pH 5.5 [to dampen the cleavage rate (38, 39)], 1 μM in vitro–reconstituted *Escherichia coli* RNase P cleaved ∼1 fM *n-Tr20* or *n-Tr20^C50U^* with similar *k*_obs_ values of 0.36 ± 0.01 or 0.26 ± 0.02 min^−1^, respectively, and different amplitudes of 98 ± 1 or 23 ± 2, respectively (Fig. 4*C* and *SI Appendix*, Fig. S8). Our finding that only one-fourth of *n-Tr20^C50U^* is cleaved in 5 mM Mg^2+^ by *E. coli* RNase P is consistent with two findings: native PAGE analysis that showed predominantly nonnative states for *n-Tr20^C50U^* in 5 mM Mg^2+^ (Fig. 3*B*) and a three- to fourfold greater apparent cleavage rate for *n-Tr20* than *n-Tr20^C50U^* in 5 mM Mg^2+^ by mouse RNase P (Fig. 2*A*).

## Discussion

Our biochemical and biophysical studies shed light on why C50U, which changes a C-G bp in *n-Tr20* to a U-G wobble bp, leads to profound neuronal dysfunction. We demonstrate here that poor processing of *n-Tr20^C50U^* by RNase P accounts for the low levels of mature and high levels of immature *n-Tr20* in the brains of B6J mice (Fig. 2). Together, our findings from thermal denaturation, native PAGE, NMR, and kinetic studies point to nonnative structure(s) associated with the C50U mutation as the basis for weak tRNA cleavage by RNase P (Fig. 4).

Why does the C50U mutation cause dramatic structural perturbations in *n-Tr20*? Instead of a largely intact native structure with replacement of a C_50_-G_64_ with a U_50_-G_64_ bp, we consider the possibility of a highly favorable realignment to a nonnative state. Like many RNAs, conformational sampling of the functional native fold as well as several inactive nonnative structures is an intrinsic, sequence context–dependent property of *n-Tr20* and other tRNAs (40). Such conformational toggling, however, poses an inherent risk for dysfunction, particularly when a mutation selectively stabilizes the nonnative state and skews the equilibrium to favor the inactive conformer. While *n-Tr20* may sample a few nonnative folds (two of which are shown in Fig. 4*A*), Mg^2+^-stabilized tertiary contacts as well as binding to RNase P nudge it toward the native state. In contrast, C50U likely promotes nonnative states with long helices, as exemplified in the nonnative 1 structure (Fig. 4*A*); here, U_50_ forms a nonnative A_42_-U_50_ bp and promotes G_43_-C_49_ and A_44_-U_48_ bp, thereby stabilizing a long, nonnative helix. Such secondary structure stability, however, comes at the cost of the canonical tRNA L-shaped tertiary structure. The cryogenic electron microscopy structures of yeast and human nuclear RNase P bound to tRNA (22–24) highlight the enzymes’ use of a shallow pocket to bind the acceptor-T arm and of π−π interactions with the G_19_-C_56_ bp of the pre-tRNA elbow to guide cleavage. The absence of this essential recognition determinant in the two predicted nonnative folds for *n-Tr20^C50U^* (Fig. 4*A*) likely accounts for its poor cleavage by RNase P. Even for the fraction that is processed, the nonnative states adopted by the mature tRNA will continue to engender adverse functional consequences (7).

While the conformational ensemble sampled by *n-Tr20* appears to be an intrinsic vulnerability, as evidenced by the C50U mutation, it may serve a biological role. *n-Tr20* could act as a precursor for generating small RNA(s), as reported in the human brain (13), and the alternate nonnative folds allow for apportioning the tRNA to this pathway instead of translation. tRNA-derived fragments play various cellular roles, including stress responses, RNA silencing, translational control, and apoptosis (1, 2, 13). In the case of a tRNA^Arg^-like *n-Tr20*, it can also act as an arginine donor for protein arginylation even after fragmentation (41). Moreover, pre-tRNA leaders and trailers have been found to accumulate as well (42). In this regard, the proximal 30-nt 5′ leader of *n-Tr20* is GU rich and is conserved completely in mammals and up to 75% in birds and reptiles (revealed by the basic local alignment search tool at National Center for Biotechnology Information). When cleaved by RNase P, this leader can potentially bind CA-rich sequences in the introns of messenger RNAs to influence alternative splicing (38) or in 3′ untranslated regions and long noncoding RNAs to affect the stability of targets RNAs (39). Thus, the equilibrium between the native and alternative folds of *n-Tr20* may regulate cleavage by RNase P to modulate the amount of free 5′ leader available for binding to other RNAs.

Other questions warrant future study. First, what causes the buildup of *n-Tr20* precursor in the postnatal mouse brain, and why does more accumulate in the cerebellum than in the cortex or hippocampus (7)? Is the native–nonnative equilibrium dependent on spatiotemporal gene expression and cellular cofactors? Second, while our studies were performed with in vitro transcripts, it would be informative to examine how other cellular events, such as cotranscriptional RNA folding and modification (8, 43, 44), affect the conformational sampling of tRNAs.

The severity of a tRNA mutation likely depends on the fractional population of mutant tRNA in its native vs. nonnative states, which is predicated on the innate propensity of the WT tRNA to embrace alternative conformations (40). While mutations that alter the stability of tRNA structures, either through modest local rearrangements or dimerization, have been documented (32, 33, 45–47), our concept of a mutation-driven large-scale restructuring that fosters alternative folds already infrequently sampled by the WT tRNA merits consideration both to better understand diseases arising from tRNA mutations [e.g., cancer, MELAS (3, 4)] and to design small molecules that steer tRNAs away from nonnative folds.

## Materials and Methods

See *SI Appendix* for details.

### Preparation of *n-Tr20* Variants and *n-Tr21*.

Sequences of *pre–n-Tr20+3′* and *–n-Tr21* were PCR amplified from C57BL/6NJ genomic DNA to include a 32-nt 5′ leader and the entire 3′ trailer upstream of the polyT. These amplicons were cloned downstream of the T7 RNA polymerase promoter in pBT7 (48). PCR was also used to generate other precursor and mature *n-Tr20* variants using *pre–n-Tr20+3′* as template. PCR with gene-specific reverse primers was used to generate templates from all these clones for runoff in vitro transcription with T7 RNA polymerase. The tRNA substrates were labeled 1) uniformly with [α-^32^P]-GTP for multiple-turnover RNase P assays and 2) at the 5′ end with [γ-^32^P]-ATP for RNase P cleavage-site mapping and for single-turnover RNase P assays.

### Partial Purification of RNase P from Mouse Cortices/Livers.

Cortices (∼1.8 g) from 4-wk-old B6J mice were lysed in extraction buffer (EB; 20 mM Tris⋅HCl, pH 8, 5 mM MgCl_2_, 14 mM β-mercaptoethanol, 0.2 mM PMSF) supplemented with 100 mM NaCl and loaded on a HiTrap DEAE-FF column (Cytiva). After elution with a linear 100–700 mM NaCl gradient, the 20 fractions were assayed for RNase P activity. The two reproducible activity peaks, P1 and P2, were pooled. P1 was further purified using a 10 to 35% (vol/vol) glycerol gradient and then concentrated approximately sevenfold using Ultra-4 centrifugal filters (3,000 nominal molecular weight limit; Amicon) to yield P1F (P1 final). Because further purification (including a glycerol gradient) led to loss of activity, P2 was concentrated ∼10-fold to yield P2F. Liver RNase P was purified using the same scheme up to the DEAE-FF elution step.

### Mouse (Multiple-Turnover) and *E. coli* (Single-Turnover) RNase P Assays.

All assays were performed at 37 °C. Purified mouse RNase P fractions were assayed in EB with 50 nM pre-tRNA and a trace amount (∼1 nM) of uniformly radiolabeled pre-tRNA. For other multiple-turnover assays, the above reactions were supplemented with 140 mM KCl. For the single-turnover assays in which only 5′-radiolabeled (∼1-fM) pre-tRNA was used, cleavage by reconstituted recombinant *E. coli* RNase P (49) was performed at pH 5.5 where the rate is slower (38, 39). After electrophoresis, gels were scanned using a Typhoon phosphorimager (GE Healthcare), and band intensities were determined using ImageQuant (GE Healthcare). In multiple-turnover assays, linear curve fits (product vs. time) were obtained using Microsoft Excel to calculate the initial velocities. In single-turnover assays, the percentage of product formed at time t (P_t_) was fit to P_t_ = P_∞_(1 − e^−kt^) using Kaleidagraph (Synergy Software).

### Thermal Denaturation of Mature *n-Tr20* and Its Mutant Derivatives.

Mature tRNAs (500 nM) in 20 mM sodium cacodylate (pH 7); 150 mM NaCl; and 0, 1, or 5 mM MgCl_2_ were analyzed in parallel using a Cary 3500 UV-visible double-beam, eight-cell Peltier spectrophotometer (Agilent). Abs_260_ (A) was monitored as the temperature (T) was increased from 20 to 95 °C at a rate of 0.5 °C/min, and T_m_ values were calculated from first-derivative plots (dA/dT vs. T).

### Native PAGE.

Mature tRNA (5 pmol) was refolded in water (95 °C for 3 min, 37 °C for 10 min) before adding 20 mM Hepes (pH 7.5), 20 mM NaCl, and either water or MgCl_2_ at the specified concentration. After a 30-min incubation at 37 °C, 11.5% (vol/vol) glycerol and trace amounts of bromophenol blue and xylene cyanol (0.01%) were added. The samples were electrophoresed at 180 V and 4 °C in a 5% (wt/vol) polyacrylamide (19:1 acrylamide/bis-acrylamide) gel made in 89 mM Tris, 89 mM boric acid, and 2 mM MgCl_2_, which were also the components in the running buffer. Gels were stained with SYBR Gold (Invitrogen) in TBE buffer for 5 min and scanned in a Typhoon phosphorimager (GE Healthcare) using the Cy2 imaging mode.

### NMR Experiments.

Diafiltration was used to buffer exchange mature *n-Tr20* variants into 15 mM sodium phosphate, pH 6.8; 25 mM NaCl; 10% (vol/vol) D_2_O; and 0, 0.5, 1, 2, 5, or 10 mM MgCl_2_. Mg^2+^-free samples contained 0.1 mM EDTA in addition. Imino ^1^H NMR spectra were acquired at 25 °C using ∼0.4 mM tRNA and a 14.1-T Bruker Avance III spectrometer equipped with an HCPN (hydrogen/carbon/phosphorus/nitrogen) cryogenic probe. All data were processed and analyzed using NMRpipe (50).

## Supplementary Material

Supplementary File

## Data Availability

All study data are included in the article and/or *SI Appendix*.
