# Supplementary material for: Structural basis for impaired 5′ processing of a mutant tRNA associated with defects in neuronal homeostasis
Source: Proc Natl Acad Sci U S A. 2022 Mar 1;119(10):e2119529119. doi: 10.1073/pnas.2119529119 (PMC8915964; doi:10.1073/pnas.2119529119)
Supplement: Supplementary File [file pnas.2119529119.sapp.pdf]

SUPPLEMENTARY INFORMATION

Structural basis for impaired 5' processing of a mutant tRNA associated with defects in neuronal homeostasis

Lien B. Lai[1,2], Stella M. Lai[1,2], Eric S. Szymanski[3,#],

Mridu Kapur[4], Edric K. Choi[1,2,#], Hashim M. Al-Hashimi[3],

Susan L. Ackerman[4,*] and Venkat Gopalan[1,2,*]

[1]Department of Chemistry & Biochemistry,

[2]Center for RNA Biology,

The Ohio State University, Columbus, OH 43210, USA

[3]Department of Biochemistry, School of Medicine,

Duke University, Durham, NC 27710, USA

[4]Howard Hughes Medical Institute, Department of Cellular and Molecular Medicine, Section of Neurobiology, University of California, San Diego, La Jolla, CA 92093, USA

* Correspondence to: Venkat Gopalan (gopalan.5@osu.edu) and Susan L. Ackerman (sackerman@health.ucsd.edu)

# Current addresses: Nymirum, Durham, NC 27713 (ES); Northwestern University, Evanston, IL 60208 (EKC)

Keywords: tRNA processing; neurodegeneration; conformational toggling

This Supplementary PDF file includes: Supplementary Text + Supplementary Figures 1-8

MATERIALS & METHODS

Preparation of *n-Tr20* variants and *n-Tr21*

Sequences of *pre–n-Tr20+3'* and *–n-Tr21* were amplified from C57BL/6NJ genomic DNA using PCR. In each case, primers were designed to include (i) 30-bp upstream flanking sequence for a 5' leader, and (ii) the entire 3' trailer sequence up to the polyT stretch that signals RNA polymerase III transcriptional termination. PCR amplicons were then inserted into the StuI site just downstream of the T7 RNA polymerase promoter in pBT7 (1); note that *in vitro* transcription (IVT) from this promoter results in two additional Gs at the 5' end of the transcript. The *pre-n-Tr20+3'* clone was used as the template for PCR-based site-directed mutagenesis to create *pre–n-Tr20* derivatives with the C50U, G64A, C50U/G64A, and C56A mutations. Mature *n-Tr20* variants were then generated by PCR-based deletion of the 5' leader. All clones were confirmed by automated sequencing at the Genome Resources Facility (OSU Comprehensive Cancer Center). PCR was also used to generate templates from the above clones for run-off IVT with T7 RNA polymerase; the forward primer F-ext anneals upstream of the T7 RNA polymerase promoter while specific reverse primers were used to either include or exclude the 3' trailer.

For multiple-turnover RNase P assays, IVT-generated unlabeled pre-tRNA substrate was spiked with a trace amount (1000 dpm/reaction) of substrate that had been uniformly labeled by IVT with [α-^{32}P]-GTP and P266L T7 RNA polymerase (2), which yielded a higher labeling efficiency than the wild type. For mapping the RNase P cleavage site and for single-turnover assays, pre-tRNA transcripts were 5'-labeled with [γ-^{32}P]-ATP and T4 polynucleotide kinase (New England Biolabs). In all instances, labeled RNAs were purified by denaturing polyacrylamide gel electrophoresis (PAGE) before use.

Partial purification of RNase P from mouse cortices/livers

Cortices from 4-week-old C57BL/6J (B6J) mice were harvested, flash frozen in liquid nitrogen, and stored at –80°C until use. After multiple pilot trials to determine the best approach for isolating native RNase P from these cortices, the following purification scheme was chosen. A motorized Dounce homogenizer was used to lyse approximately 1.8 g of tissue in 20 mL ice-cold extraction buffer (EB: 20 mM Tris-HCl, pH 8 at 23°C; 5 mM $MgCl_2$; 14 mM β-mercaptoethanol; 0.2 mM PMSF) supplemented with 100 mM NaCl (EB100). Cellular debris was removed by centrifugation at 21,000 x *g* for 30 min at 4°C. After passing through a 0.45-μm syringe filter, the lysate was loaded onto a 1-mL HiTrap DEAE-FF column (Cytiva) pre-equilibrated with EB100. Using an ÄKTA FPLC (Cytiva), the column was washed with 8 mL EB100 and bound species subsequently eluted using a linear 100–700 mM NaCl (in EB) gradient in 20 x 0.5-mL fractions. Each fraction was then assayed for RNase P activity with *pre–n-Tr20* and *pre–n-Tr21* (Fig. 1 *A* and B) to determine the peaks of activity (Fig. 1 *C* and *D*). Fractions #7–12 were pooled corresponding to peak 1 (P1) and #13–20 to P2 (Fig. 1 *C* and *D*). P1 was further purified using a 10–35% (v/v) glycerol gradient (in EB): 1.25 mL P1 was loaded on each of two 10-mL gradients and spun at 200,000 x *g* for 18 h at 4°C using a Beckman ultracentrifuge. Each gradient was aliquoted from top to bottom into 22 x 0.5-mL fractions. After assaying for RNase P activity (Fig. 1 *E* and *F*) with *pre–n-Tr20* and *pre–n-Tr21*, peak fractions #14–16 were pooled and concentrated ~7-fold using Ultra-4 centrifugal filters (3,000 NMWL, Amicon) to yield P1F (final P1). Because further purification (including a glycerol gradient) led to loss of activity, P2 was concentrated ~10-fold to yield P2F.

For purification of RNase P from mouse livers (*SI Appendix,* Fig. S3), we used the same scheme as that used for the mouse brain except that we only followed the steps until the DEAE-FF elution.

Mouse RNase P assays

DEAE-purified fractions were assayed in EB, 50 nM pre-tRNA, and a trace amount (~1 nM) of uniformly radiolabeled pre-tRNA; the NaCl concentration in each reaction varied (30–210 mM) because each fraction contained a different amount of NaCl due to the gradient employed for elution. For all other multiple-turnover assays, the above reactions were supplemented with 140 mM KCl. All reactions were incubated in either a 37°C water bath or a thermocycler for the indicated period. Time-course reactions were set up in a large volume and aliquots withdrawn at specific time intervals (typically every 5 min). We ensured that total cleavage did not exceed 25% of the substrate. The reactions/aliquots were immediately terminated by adding 1–3 volumes of quench dye [7 M urea, 1 mM EDTA, 0.05% (w/v) bromophenol blue, 0.05% (w/v) xylene cyanol, 10% [v/v] phenol]. Samples were then electrophoresed on 8% (w/v) acrylamide/7 M urea gels, which were subsequently scanned using a Typhoon phosphorimager (GE Healthcare). ImageQuant (GE Healthcare) was used to determine band intensities. The amount of product generated as a function of time was then plotted and the data fit to a straight line in Microsoft Excel to obtain the initial velocity. All linear curve fits had correlation coefficients (R^2) ≥ 0.96. For rate determination, all initial velocity measurements were performed when P1F and P2F were less than a week old to ensure that they were highly active. Although all three assay replicates were also completed on the same day to minimize variability (Fig. 2), we verified that trends were reproducible across independent preparations of mouse brain RNase P.

Thermal denaturation of mature *n-Tr20* and its mutant derivatives

One-mL solutions containing 500 nM tRNA in 20 mM sodium cacodylate, pH 7; 150 mM NaCl; and either 0, 1, or 5 mM $MgCl_2$ were prepared and transferred to rectangular semi-micro quartz spectrophotometer cuvettes (FireflySci, Staten Island, NY) with 1-cm path lengths and polytetra-fluoroethylene stoppers, which minimized sample loss by evaporation. Because the pKa of

cacodylate (~6.3) is independent of temperature, all thermal denaturation profiles were obtained in sodium cacodylate instead of Tris, the buffering agent used during enzyme purification and characterization. Multiple samples were analyzed in parallel using a Cary 3500 ultraviolet (UV)-visible double beam, multi-cell Peltier spectrophotometer (Agilent) equipped with eight cuvette positions, which increased experiment throughput and permitted the comparison of samples under identical conditions. Absorbance at 260 nm was monitored as the temperature was increased from 20°C to 95°C at a rate of 0.5°C/min.

T_m values were calculated from first-derivative plots (dA/dT vs. T, where A is absorbance and T is temperature) generated using the *Analysis* function of the Cary UV Workstation Thermal program (Agilent) and a low/high calculation limit of 55–80°C. Mean and standard deviation values were calculated from three independent replicates of each tRNA in each $MgCl_2$ concentration. Microsoft Excel was used to normalize thermal denaturation curves and to smooth the first derivative (using the moving averages function and an interval of 5), while Kaleidagraph (Synergy Software) was used to plot and overlay the resultant data (Fig. S4).

Although first-derivative analyses of thermal denaturation curves offer an expedient approach to determining T_m values, fitting the data to the van 't Hoff equation allows for independent cross-validation of these T_m values. T_m values calculated using the first-derivative approach were in good agreement with those obtained using the van 't Hoff equation (3). Note that the tRNAs used in ref. 3 contained a 1-nt leader that was absent in all *n-Tr20* variants used here.

Native polyacrylamide gel electrophoresis (PAGE)

Five pmol of mature tRNA was incubated in water at 95°C for 3 min and at 37°C for 10 min before 2 μL 5X buffer [100 mM HEPES, pH 7.5 at 22°C; 100 mM NaCl] and either water or $MgCl_2$ were added to a final volume of 10 μL with to achieve the desired $MgCl_2$ concentration. The sample

was then incubated at 37°C for an additional 30 min. Subsequently, 3 μL loading buffer [50% (v/v) glycerol, 0.05% (w/v) bromophenol blue, 0.05% (w/v) xylene cyanol] was added and the samples were kept at 37°C until loading. The same buffer (89 mM Tris, 89 mM boric acid, 2 mM $MgCl_2$) was used to prepare 5% (w/v) polyacrylamide (19:1 acrylamide:bis-acrylamide) gels and used as the running buffer for electrophoresis. Gels (17 cm width x 15 cm height) were electrophoresed at 180 V and 4°C until the bromophenol blue dye reached the bottom. Gels were stained with SYBR Gold (Invitrogen) in TBE buffer for 5 min and then scanned using a Typhoon phosphorimager (GE Healthcare) and the Cy2 imaging mode.

NMR experiments

Diafiltration was used to buffer exchange mature *n-Tr20* variants into 15 mM sodium phosphate, pH 6.8; 25 mM NaCl; 10% (v/v) D_2O; and 0, 0.5, 1, 2, 5, or 10 mM $MgCl_2$; Mg^{2+}–free samples contained 0.1 mM EDTA in addition. Imino ^1H NMR spectra were acquired at 25°C using ~0.4 mM tRNA and a 14.1 Tesla Bruker Avance III spectrometer equipped with an HCPN cryogenic probe. All data were processed and analyzed using NMRpipe (4).

Single-turnover experiments with *E. coli* RNase P

Since we expected *in vitro* reconstituted *E. coli* RNase P to cleave *pre–n-Tr20* too rapidly at near-neutral pH, we resorted to an assay pH of 5.5 where the rate is lower (5, 6) and assays can be performed manually. For these single-turnover studies, the *in vitro* transcribed *E. coli* RNase P RNA was first refolded. The RNA was incubated in water at 50°C for 30 min and at 37°C for 10 min, followed by addition of an equal volume of 2X binding buffer [2X BB: 40 mM cacodylic acid, 800 mM ammonium acetate, 10 mM magnesium acetate, 10% (v/v) glycerol, 0.02% (v/v) IGEPAL; the solution was pH-adjusted to 5.5 with HCl] and an incubation at 37°C for 30 min. The refolded RNA was then mixed with *E. coli* RNase P protein (final RNA:protein ratio of 1:2.5) and the mixture

was incubated at 37°C for 5 min to promote assembly. To determine the k_{obs} at 37°C, 2000 dpm of 5'-radiolabeled *pre–n-Tr20* or *pre–n-Tr20*^{C50U} (~1 fM) was added to the reaction (without unlabeled pre-tRNA) and aliquots were withdrawn at intervals and immediately quenched with 3 volumes of 7 M urea dye [7 M urea, 1 mM EDTA, 0.05% (w/v) bromophenol blue, 0.05% (w/v) xylene cyanol, 10% (v/v) phenol]. The time intervals were established in pilot trials to allow for near-complete conversion of substrate to product. The quenched reactions were subsequently electrophoresed on 8% (w/v) acrylamide/7 M urea gels. These gels were scanned using a Typhoon phosphorimager (GE Healthcare) to help quantitate the substrate and product amounts. The percent of product formed at time t (P_t) was fit to $P_t = P_\infty(1 - e^{-kt})$ using Kaleidagraph (Synergy Software). The individual curve-fit errors for k_{obs} and amplitudes are provided in the data shown for each trial (*SI Appendix,* Fig. S8). All curve fits had correlation coefficients ≥ 0.98. Three replicates were performed to obtain the mean and standard deviation values listed in Figure 4C.

REFERENCES

1. H. Y. Tsai, L. B. Lai, V. Gopalan, A modified pBluescript-based vector for facile cloning and transcription of RNAs. *Anal Biochem* **303**, 214-217 (2002).
2. S. Lyon, V. Gopalan, A T7 RNA polymerase mutant enhances the yield of 5'-thienoguanosine-initiated RNAs. *Chembiochem* **19**, 142-146 (2018).
3. S. M. Lai (2017) Defective tRNA processing by RNase P contributes to neurodegeneration in mice. M.S. Thesis, The Ohio State University, Columbus, OH.
4. F. Delaglio et al., NMRPipe: a multidimensional spectral processing system based on UNIX pipes. *J Biomol NMR* **6**, 277-293 (1995).
5. J. Hsieh, C. A. Fierke, Conformational change in the Bacillus subtilis RNase P holoenzyme--pre-tRNA complex enhances substrate affinity and limits cleavage rate. *RNA* **15**, 1565-1577 (2009).
6. L. Sun, F. E. Campbell, N. H. Zahler, M. E. Harris, Evidence that substrate-specific effects of C5 protein lead to uniformity in binding and catalysis by RNase P. *EMBO J* **25**, 3998-4007 (2006).
7. L. B. Lai et al., Discovery of a minimal form of RNase P in Pyrobaculum. *Proc Natl Acad Sci USA* **107**, 22493-22498 (2010).
8. V. Gopalan, A. D. Baxevanis, D. Landsman, S. Altman, Analysis of the functional role of conserved residues in the protein subunit of ribonuclease P from Escherichia coli. *J Mol Biol* **267**, 818-829 (1997).
9. L. A. Kirsebom, S. Trobro, RNase P RNA-mediated cleavage. *IUBMB Life* **61**, 189-200 (2009).
10. Y. Wang, G. Han, X. Jiang, T. Yuwen, Y. Xue, Chemical shift prediction of RNA imino groups: application toward characterizing RNA excited states. *Nat Commun* **12**, 1595 (2021).

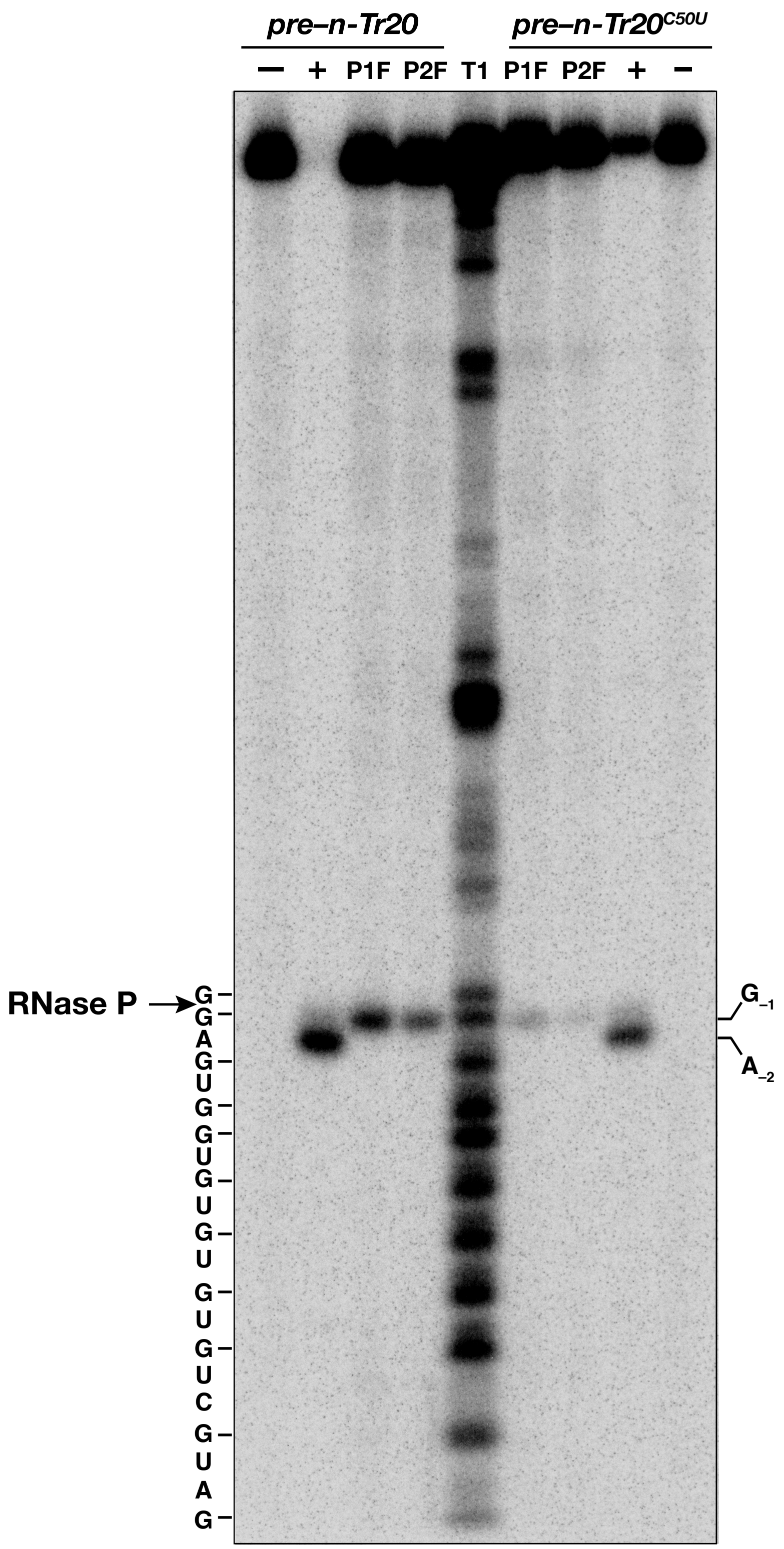

Fig. S1. Mouse brain RNase P cleaves both *pre–n-Tr20* and *pre–n-Tr20*^{C50U} at the correct site.

For this cleavage-site mapping experiment, 5'-[32P]-labeled *pre–n-Tr20* and *pre–n-Tr20*^{C50U} were

used (see (7) for another example). Lanes: –, negative control containing substrate but no enzyme; +, positive control with substrate and *in vitro* reconstituted *Escherichia coli* RNase P (8); P1F and P2F, the two peaks of mouse brain RNase P activity; T1, a ladder generated by partial digestion of denatured *pre–n-Tr20* with RNase T1, a nuclease that cleaves 3' to G residues. Following RNase T1 digestion, cleavage products were treated with T4 polynucleotide kinase to remove their 3'-phosphate and normalize their migration with the RNase P-cleaved 5' leader that contains a 3'-OH. The 5' leader sequence and the RNase P cleavage site (arrow) are provided to the left of the gel image, with G nucleotides matched to those observed in the T1 ladder. Note that while the 5' leaders of both *pre–n-Tr20* and *pre–n-Tr20*^{C50U} cleaved by P1F and P2F match the correct product (i.e., cleavage between G_{-1} and G_{+1}), the leader generated by *E. coli* RNase P corresponds to mis-cleavage between G_{-1} and A_{-2}, likely because *pre–n-Tr20* and *pre–n-Tr20*^{C50U} have a G_{-1} (a negative determinant) and lack a 3'-CCA (a positive determinant for bacterial RNase P) (9). This mis-cleavage (i.e., single-nt shift) by *E. coli* RNase P is evident only when employing long gels such as the one shown here and not in any of the assay gels used in this study.

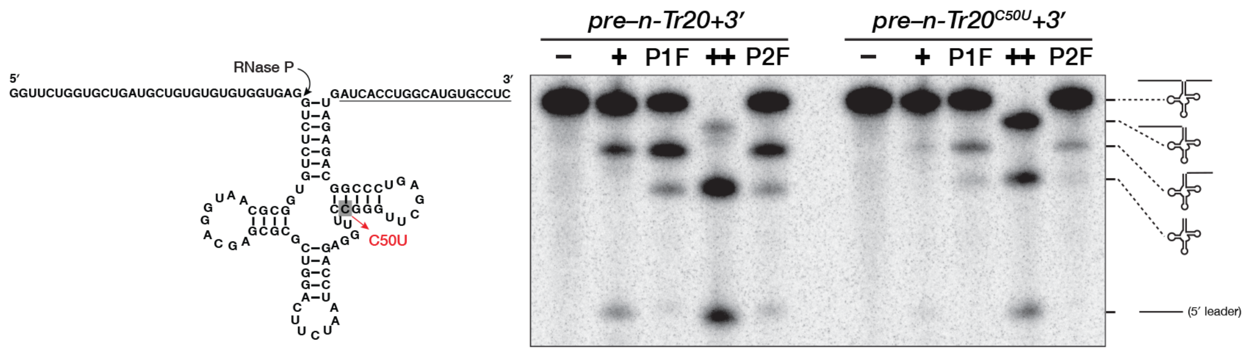

Fig. S2. Mouse brain RNase P can cleave *pre–n-Tr20+3′* and *pre–n-Tr20^{C50U}+3′* with a 32-nt 5' leader and a 19-nt 3' trailer (underlined in the *pre–n-Tr20* structure on the left). Lanes: –, negative control containing substrate but no enzyme; +, positive control with the pre-tRNA+3' substrate and *in vitro* reconstituted *E. coli* RNase P (8); ++, positive control with the pre-tRNA (no trailer) substrate and *in vitro* reconstituted *E. coli* RNase P. The two positive controls provide markers for the various unprocessed and processed tRNA species that are schematized to the right of the assay gel. Note that despite the fortuitous presence of a 3' processing activity in fractions enriched for RNase P, no intermediate species containing a 5' leader but lacking a 3' trailer was observed under these assay conditions, revealing a 5'-before-3' cleavage order.

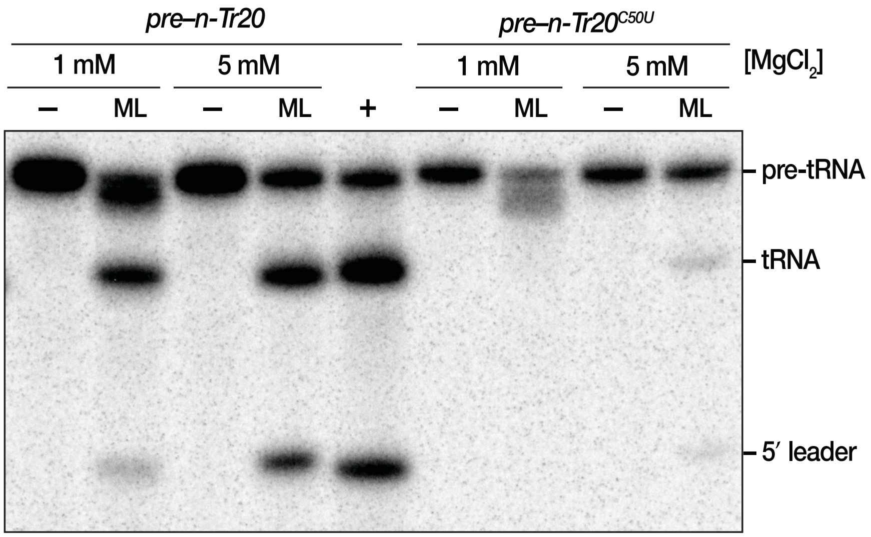

Fig. S3. *Pre–n-Tr20*^{C50U} is also poorly processed by mouse liver RNase P in 1 or 5 mM $MgCl_2$. The enzyme was purified using DEAE (anion-exchange) chromatography and one fraction within peak 1 (P1) was assayed. Lanes: –, negative control containing substrate but no enzyme; +, positive control with substrate and *in vitro* reconstituted *E. coli* RNase P (8); ML, mouse liver RNase P.

A

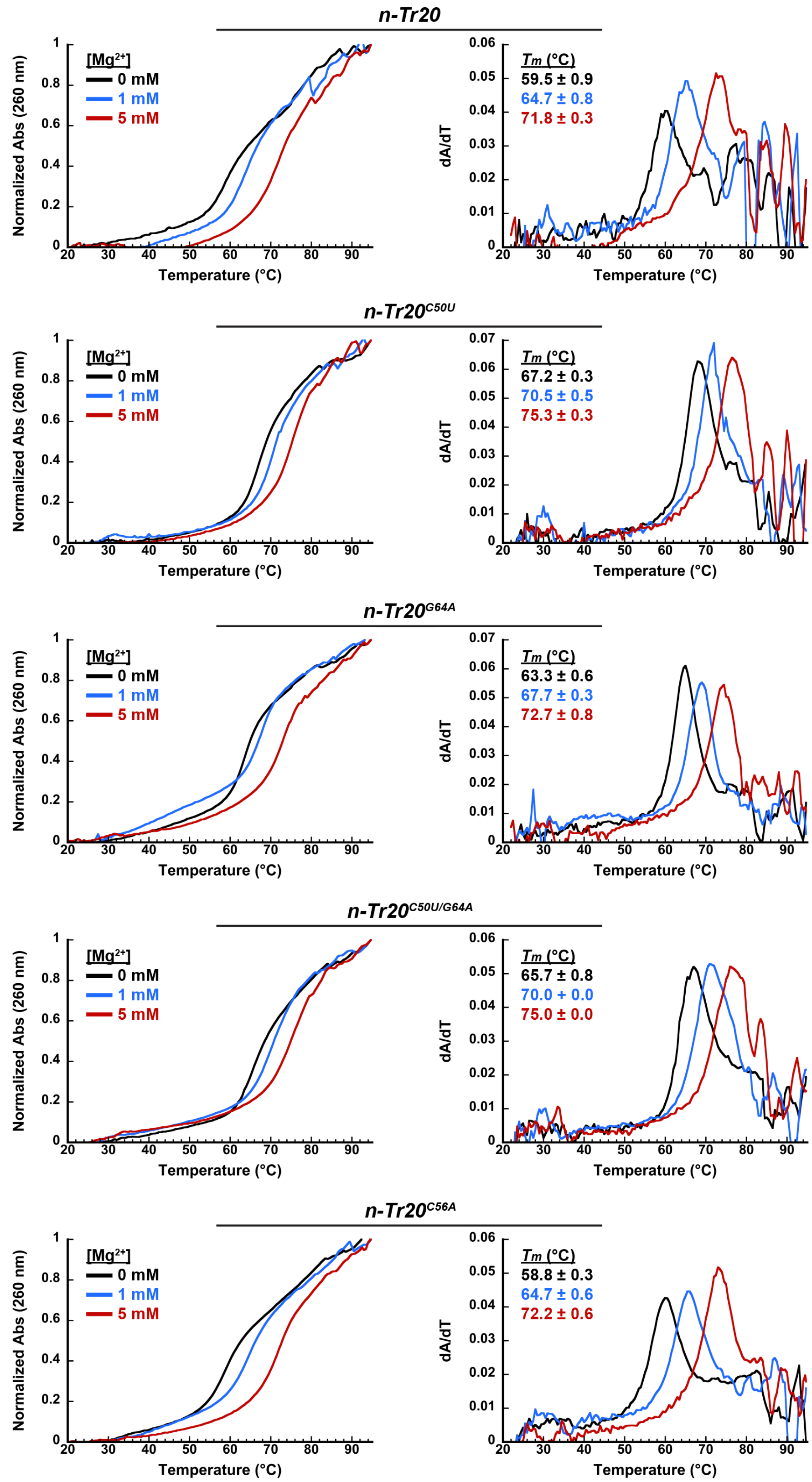
n-Tr20
Normalized Abs (260 nm)
[Mg2+]
0 mM
1 mM
5 mM
Temperature (°C)
dA/dT
Tm (°C)
59.5 ± 0.9
64.7 ± 0.8
71.8 ± 0.3
n-Tr20C50U
[Mg2+]
0 mM
1 mM
5 mM
Tm (°C)
67.2 ± 0.3
70.5 ± 0.5
75.3 ± 0.3
n-Tr20G64A
[Mg2+]
0 mM
1 mM
5 mM
Tm (°C)
63.3 ± 0.6
67.7 ± 0.3
72.7 ± 0.8
n-Tr20C50U/G64A
[Mg2+]
0 mM
1 mM
5 mM
Tm (°C)
65.7 ± 0.8
70.0 + 0.0
75.0 ± 0.0
n-Tr20C56A
[Mg2+]
0 mM
1 mM
5 mM
Tm (°C)
58.8 ± 0.3
64.7 ± 0.6
72.2 ± 0.6

B

	T_m (°C)		
**tRNA**	**0 mM $MgCl_2$**	**1 mM $MgCl_2$**	**5 mM $MgCl_2$**
n-Tr20	59.5 ± 0.9	64.7 ± 0.8	71.8 ± 0.3
***n-Tr20**^{C50U}*	67.2 ± 0.3	70.5 ± 0.5	75.3 ± 0.3
***n-Tr20**^{G64A}*	63.3 ± 0.6	67.7 ± 0.3	72.7 ± 0.8
***n-Tr20**$^{C50U/G64A}$*	65.7 ± 0.8	70.0 ± 0.0	75.0 ± 0.0
***n-Tr20**^{C56A}*	58.8 ± 0.3	64.7 ± 0.6	72.2 ± 0.6

Fig. S4. Thermal denaturation analysis of mature *n-Tr20* and its mutant derivatives in 0, 1, or 5 mM $MgCl_2$. (*A*) Plots of normalized absorbance at 260 nm versus temperature (*left panels*), and their corresponding first-derivative plots (*right panels*) for each tRNA at all three $MgCl_2$ concentrations. (*B*) T_m values determined for each tRNA at all three $MgCl_2$ concentrations. Note that all mutants, except for C56A, are more stable than *n-Tr20*. Mean and standard deviation values were calculated from three independent thermal melts.

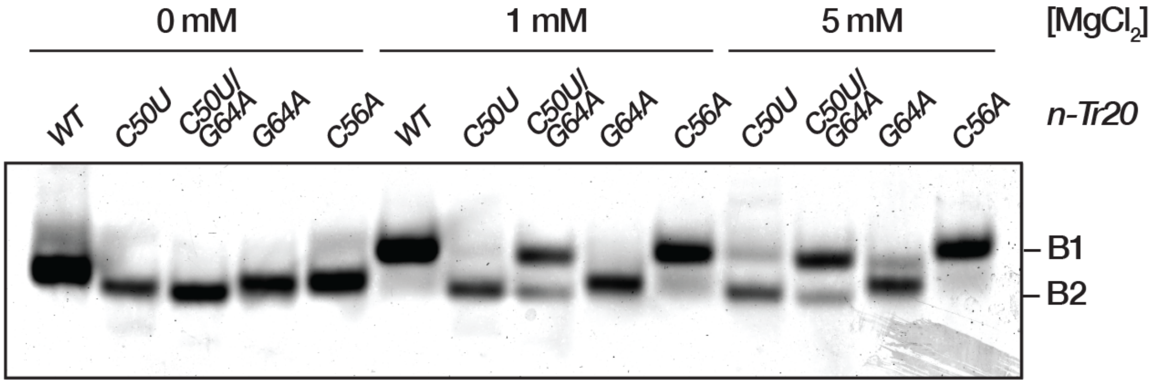

Fig. S5. Native PAGE analyses reveal Mg^{2+}-dependent tRNA conformational changes among the mature *n-Tr20* variants. In 0 mM $MgCl_2$, all tRNAs migrated as B2. In 1 mM $MgCl_2$, nearly all wild type (WT) and *n-Tr20*^{C56A} shifted to B1, but the latter had residual B2. Very little of *n-Tr20*^{C50U} migrated as B1 even in 5 mM $MgCl_2$, while *n-Tr20*$^{C50U/G64A}$ responded more acutely to increasing [$MgCl_2$] with most of it in B1 by 5 mM $MgCl_2$. In contrast, *n-Tr20*^{G64A} seemed to migrate as two bands that are in between B1 and B2, with the slower migrating band becoming prominent with increasing [$MgCl_2$].

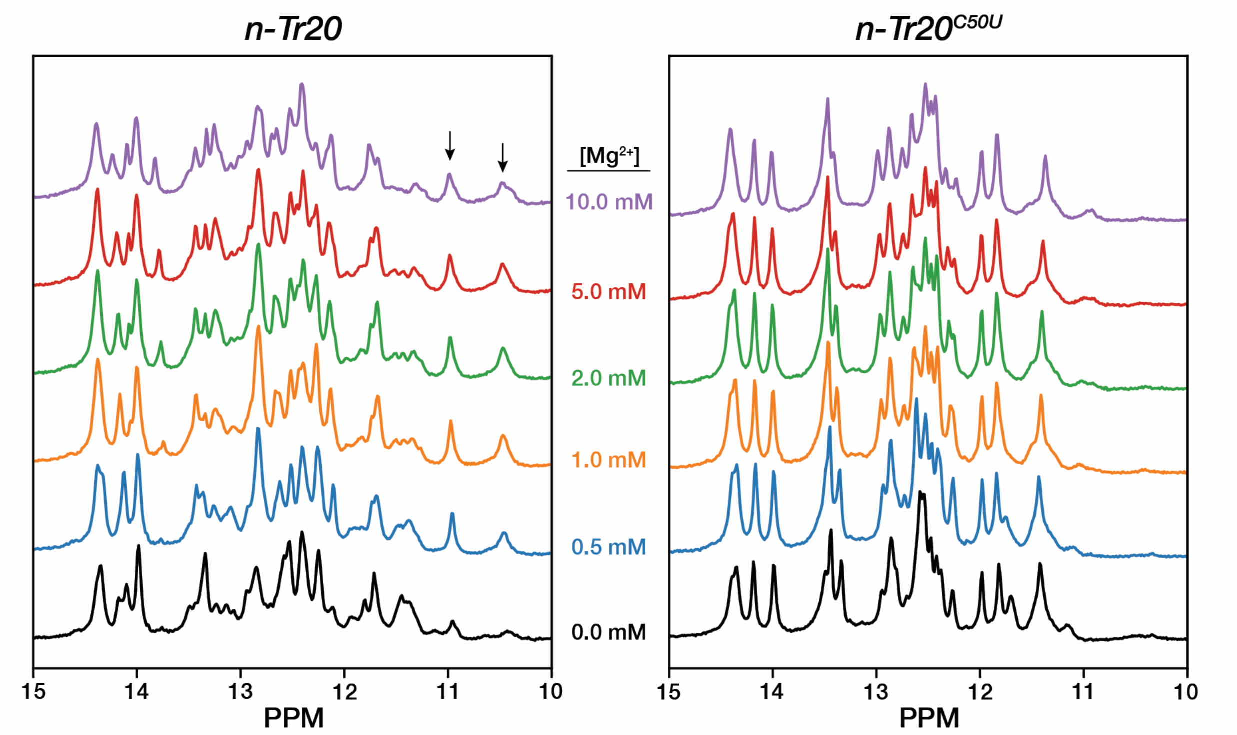

Fig. S6. Imino proton region (10) of the ^{1}H NMR spectra of mature *n-Tr20* and *n-Tr20*^{C50U} as a function of increasing [$MgCl_2$]. Black arrows indicate the two signature upfield peaks that are likely associated with tertiary interactions.

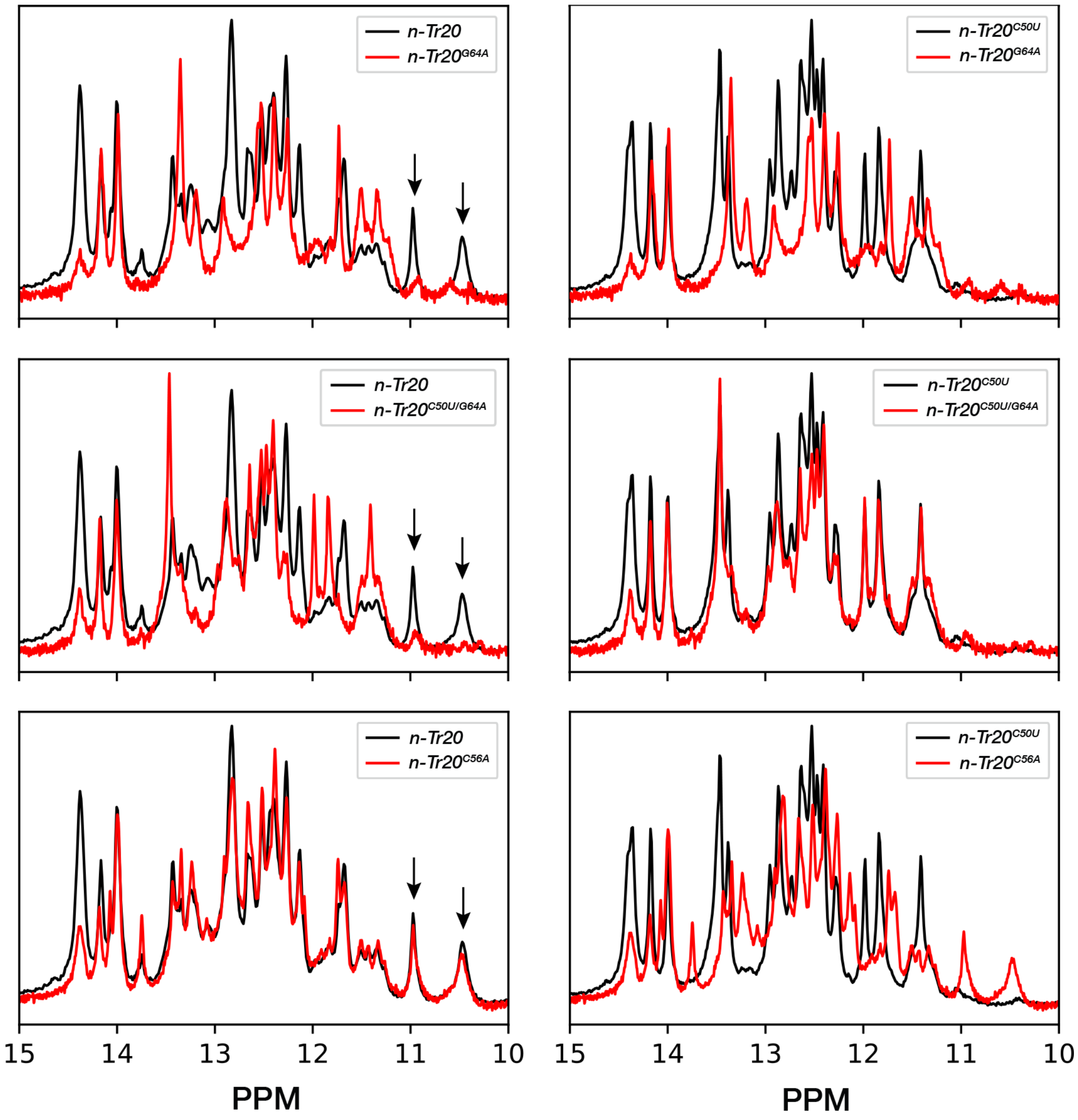

Fig. S7. Comparison of the imino proton region of the ^{1}H NMR spectra of mature *n-Tr20* or *n-Tr20*^{C50U} versus other *n-Tr20* mutant derivatives in 1 mM Mg^{2+}. *n-Tr20*^{G64A} (*top*), *n-Tr20*$^{C50U/G64A}$ (*middle*), and *n-Tr20*^{C56A} (*bottom*) are superimposed on *n-Tr20* (*left*) and *n-Tr20*^{C50U} (*right*). Note the similarity between *n-Tr20* and *n-Tr20*^{C56A} and between *n-Tr20*^{C50U} and *n-Tr20*$^{C50U/G64A}$, as well as the distinctive features of *n-Tr20*^{G64A}. Black arrows indicate the two signature upfield peaks that are likely associated with tertiary interactions.

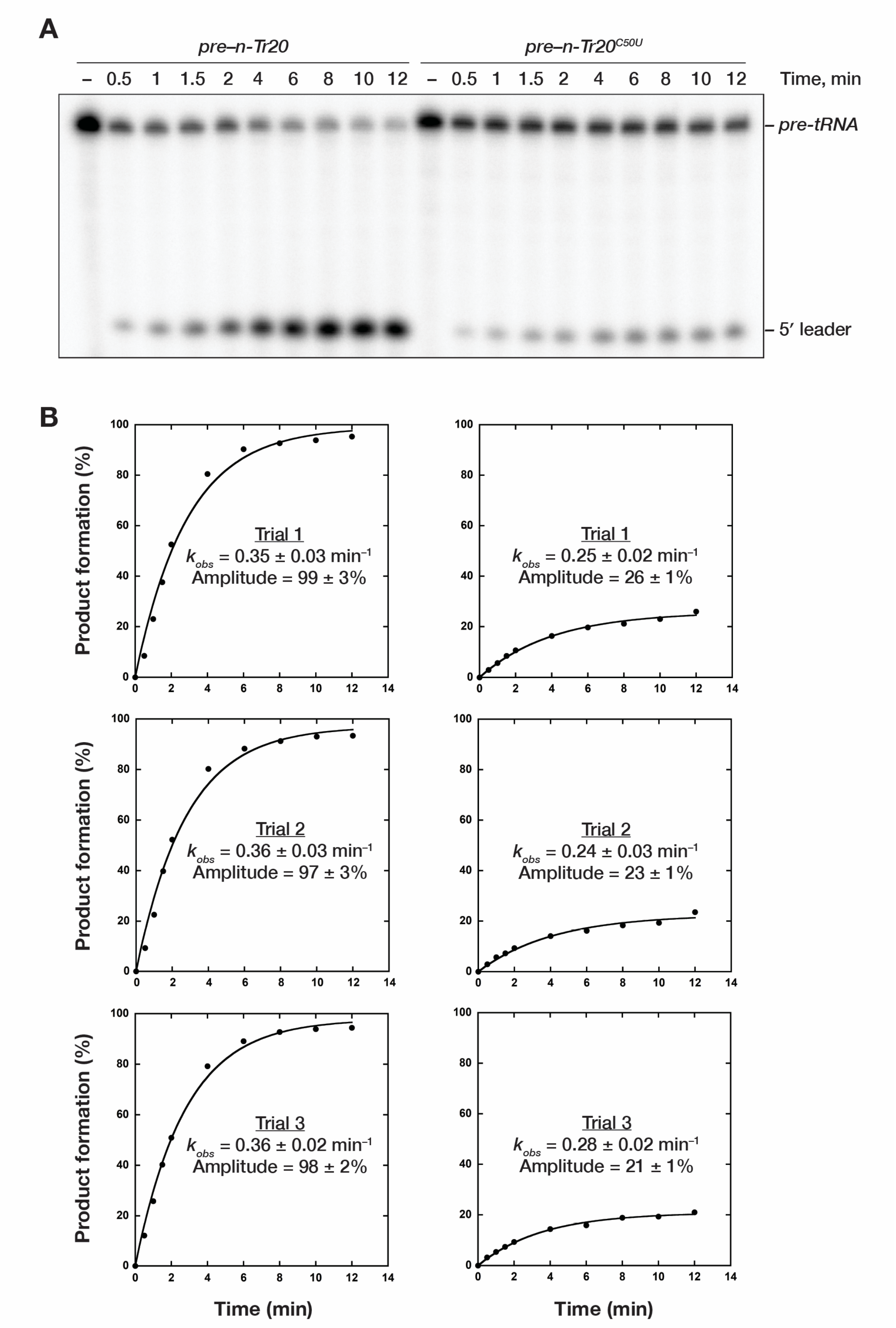

Fig. S8. Single-turnover cleavage of *pre–n-Tr20* or *pre–n-Tr20*^{C50U} by *E. coli* RNase P. (*A*) Gel image corresponding to the Trial 1 time-course study with both substrates. –, a negative control

with substrate but no enzyme. Because the pre-tRNA substrates were radiolabeled at the 5' end, only the unprocessed substrate and the 5' leader were detected. (*B*) Plots of 5' leader (product) formation at each time point are shown for the three trials with each substrate (*left*, *pre–n-Tr20*; *right*, *pre–n-Tr20*^{C50U}). The k_{obs} and amplitude of each trial are displayed here, and the mean and standard deviation values calculated from the three replicates for each substrate are listed in Figure 4C.
